# A decision-support tool for management of advanced epithelial ovarian cancer in a single centre in India (CT PAUSE Nomogram): a prospective Study (2022–2024)

**DOI:** 10.1016/j.lansea.2026.100768

**Published:** 2026-04-29

**Authors:** Angelin Grace Jeslin, Shobiga Natarajan, Sneha Hiriyanna, Renu Ninan, Goldwin Helan Cecil, Betty Simon, Anu Eapen, Vinotha Thomas, Anitha Thomas, Kripa M. Varghese, Anjana Joel, Ashish Singh, Reka Karuppusami, Anuradha Chandramohan

**Affiliations:** a Abdominal Imaging Unit, Department of Radiology, Christian Medical College Vellore, India; b Department of Gynaecological Oncology, Christian Medical College Vellore, India; c Department of Pathology, Christian Medical College Vellore, India; d Department of Medical Oncology, Christian Medical College Vellore, India; e Department of Biostatistics, Christian Medical College Vellore, India

**Keywords:** Ovarian cancer, Cytoreductive surgery, PAUSE score, CT, Peritoneal metastases, Surgical triage, Peritoneal cancer index

## Abstract

**Background:**

Accurate preoperative imaging is essential in advanced epithelial ovarian cancer, where complete cytoreduction remains the strongest prognostic factor. This study evaluates the CT PAUSE score—a structured, domain-based reporting tool—for its utility in surgical planning and multi-disciplinary team (MDT) decision-making.

**Methods:**

In this prospective cross-sectional study between September 2022 to February 2024, 124 patients with FIGO stage III/IV ovarian cancer underwent 175 contrast-enhanced CT scans. PAUSE components—Peritoneal Cancer Index (PCI), Ascites/abdominal wall disease, Unfavourable sites, Small bowel/mesenteric disease, and Extra-peritoneal metastases—were prospectively scored during evaluation. Interobserver agreement was assessed in a subset of 30 cases.

**Findings:**

MDT triage using PAUSE resulted in a complete cytoreduction rate of 89.3%. A simplified nomogram based on upper abdominal disease volume showed discriminatory ability (area under the curve (AUC) [95% CI] = 0.820 [0.740–0.880]) and could be an alternative to the full radiological PCI-based nomogram (AUC) [95% CI] = 0.763 [0.677–0.835]), in busy clinical settings. Interobserver agreement was substantial for both nomogram scores, with higher reliability observed for the score derived from the upper abdominal disease–based nomogram (intraclass correlation coefficient (ICC) [95% CI] = 0.710 [0.582–0.825] versus 0.627 [0.460–0.778]; p < 0.001).

**Interpretation:**

CT PAUSE provides a structured approach to radiological reporting and may support more consistent MDT discussions and surgical triage in advanced ovarian cancer. Its performance in this cohort might suggest potential for integration into clinical workflows, pending further validation.

**Funding:**

There was no funding source for this study.

**Research in context**

**Evidence before this study**

Ovarian carcinoma commonly presents with advanced peritoneal dissemination, where complete cytoreduction is the strongest prognostic factor. Accurate preoperative imaging is therefore important, yet variability in CT reporting limits its utility for surgical planning. Recent joint recommendations from the European Society of Gastrointestinal and Abdominal Radiology (ESGAR), the European Society of Uro-Radiology (ESUR), the Peritoneal Surface Oncology Group International (PSOGI), and the European Association of Nuclear Medicine (EANM) advocate structured reporting tools such as PROMISE and PAUSE to increase consistency and to support multi-disciplinary team decision making. The CT-based PAUSE tool integrates radiological peritoneal cancer index (rPCI), ascites, abdominal wall involvement, unfavourable sites, small bowel/mesenteric disease, and extra-peritoneal metastases, but prospective evidence in ovarian cancer remains scarce.

**Added value of this study**

In a prospective cohort of 124 patients with FIGO stage III/IV ovarian cancer, we applied the CT PAUSE score during routine multi-disciplinary team (MDT) planning. In this cohort, use of PAUSE was associated with MDT surgical triage and a complete cytoreduction rate of 89.3%. We also developed a simplified nomogram based on upper abdominal disease volume, which showed discriminatory performance with AUC of 0.820 and higher interobserver agreement than the rPCI-based model. This simplified approach may provide an alternative in busy clinical settings.

**Implications of all the available evidence**

These findings indicate that CT PAUSE could have a role as a structured adjunct within routine staging workflows for advanced ovarian cancer. By standardising radiological assessment, CT PAUSE may facilitate more consistent multi-disciplinary team discussions and improve alignment between radiologists and surgeons regarding disease extent and surgical expectations. The simplified nomogram showed acceptable discriminatory performance in this cohort and could serve as an alternative approach to structured imaging assessment, particularly in high-volume clinical settings.

## Introduction

Ovarian carcinoma presents at an advanced stage in 60–80% of patients,^1^ often with extensive peritoneal dissemination that adversely affects prognosis.^2^^,^^3^ The key component of ovarian cancer management is cytoreductive surgery.^4^ Complete cytoreduction (CC0) or surgical removal of all visible disease has been shown to be consistently associated with improved overall and disease free survival outcomes.^4^, ^5^, ^6^, ^7^, ^8^ Similarly, heated intraperitoneal chemotherapy (HIPEC) in a setting of interval cytoreduction improved survival outcomes.^9^ Accurate preoperative assessment of peritoneal disease burden is crucial for predicting feasibility of complete cytoreduction and for guiding optimal treatment strategies. Depending on the local expertise and resource setting, CT, MRI, PET-CT and PET-MRI are imaging modalities that are used for staging and preoperative assessment of peritoneal disease.^10^^,^^11^ Contrast-enhanced CT remains the preferred first imaging modality and the workhorse for staging ovarian cancer.^11^

There is wide variability in radiological reporting styles for ovarian cancer and this can hinder effective communication and surgical planning.^12^^,^^13^ Standardising radiological evaluation is therefore critical for improving consistency, enhance multi-disciplinary team (MDT) decision-making, and align expectations between radiologists and surgeons.^14^ There are been several previous attempts to provide structured reporting methods and review areas such as peritoneal cancer index (PCI), CT resectability scores such as R-score or ESMO-ESGO resectability criteria, acronyms like PAUSE, and BUMPy.^5^^,^^15^, ^16^, ^17^, ^18^, ^19^, ^20^, ^21^ PAUSE is an acronym that stands for *P*eritoneal Cancer Index (PCI), *A*scites and abdominal wall involvement, *U*nfavourable sites, *S*mall bowel and mesenteric disease, and *E*xtra-peritoneal disease and BUMPy stands for *B*owel, *U*pper abdomen, *M*esentery in *P*eritoneal disease.^20^, ^21^, ^22^ Recent joint recommendations from ESGAR, ESUR, PSOGI, and EANM on the imaging of peritoneal metastases from ovarian and colorectal cancer recommend the use of a structured reporting tool, such as PROMISE and PAUSE.^23^ PAUSE has been adopted in several specialist peritoneal surface malignancy centres, particularly for colorectal peritoneal metastases and peritoneal mesothelioma.^24^, ^25^, ^26^ However, its role in ovarian cancer management and evidence from real-world clinical use have not been described. In this prospective cohort of patients with ovarian malignancy, PAUSE was incorporated into routine MDT workflow to explore its utility in staging and cytoreductive surgical triage.

## Methods

**Fig. 1:**
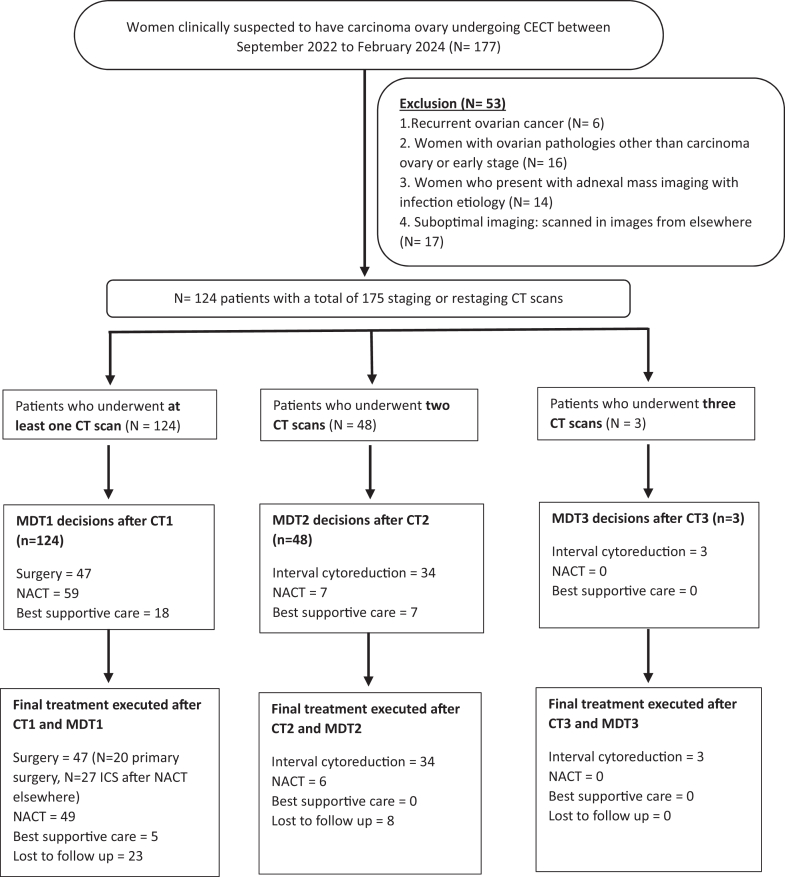
STROBE-compliant (Strengthening the Reporting of Observational Studies in Epidemiology) flow diagram illustrating recruitment, inclusion, and follow-up of the study cohort.

This prospective cross-sectional study was conducted at Christian Medical College Vellore, a tertiary care academic institution with a specialised gynaecological oncology multi-disciplinary team (MDT) located in Vellore, Tamil Nadu, India. We included consecutive patients diagnosed with advanced epithelial ovarian cancer (FIGO stage III and above) who underwent either primary or interval cytoreductive surgery between September 1, 2022, and February 28, 2024. The STROBES-compliant patient flow diagram (Fig. 1) outlines the inclusion and exclusion criteria.

All patients underwent a contrast-enhanced CT scan of the thorax, abdomen and pelvis. Positive oral contrast was administered regularly. The CT protocol included the administration of 70–100 mL of intravenous non-ionic iodinated contrast media followed by a 20 mL saline flush at a flow rate of 5 mL/s. Thin-section axial images were acquired and reconstructed into 2 mm-thick sections in axial and coronal planes for radiologists' reading in the Picture Archiving and Communication System (PACS). Two radiologists reviewed CT images from initial staging and restaging scans. All consecutive CT scans of patients clinically suspected to have ovarian cancer were read, but only those with histopathologically confirmed epithelial ovarian carcinoma, stage III or higher, were included in the final analysis.

**Fig. 2:**
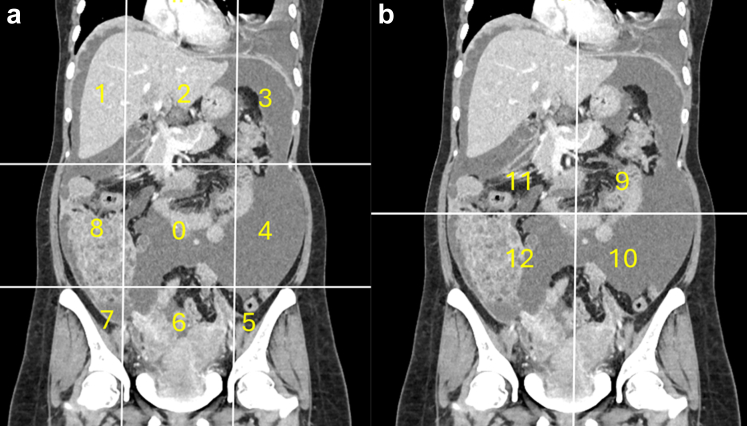
Calculation of radiological peritoneal cancer index (rPCI) adapted from Sugarbaker et al. (1). (a) Coronal CT showing regions 0–8. Vertical lines are drawn along the right and left midclavicular line and the horizontal lines along the costal margin and the iliac crests. (b) Coronal CT showing regions 9 to 12. The vertical and the horizontal lines are through the umbilicus divide the small bowel and mesentery into proximal and distal jejunal and ileal regions.

**Supplementary Figs. S1:**
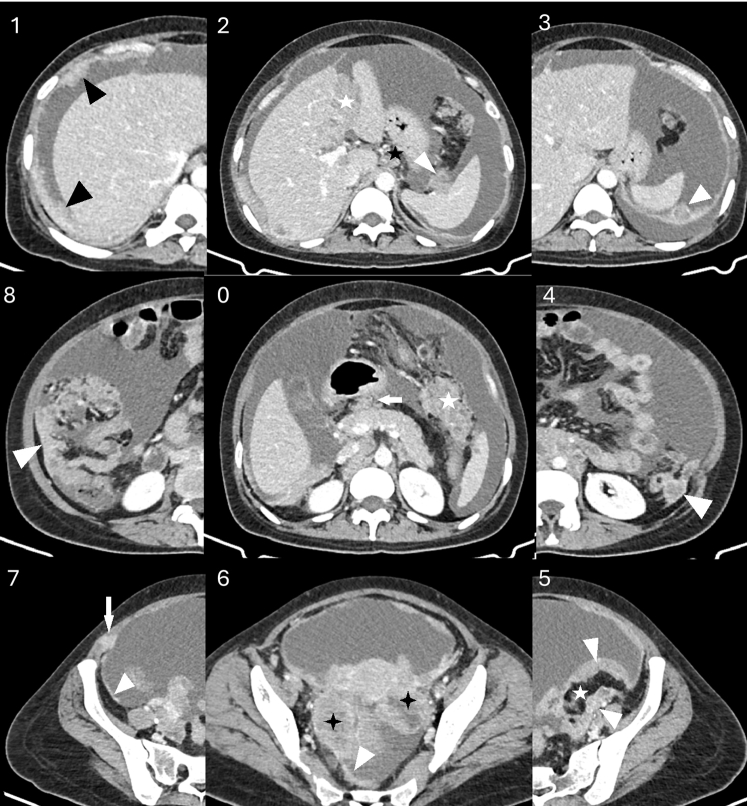
Panel of contrast-enhanced axial CT images depicting disease (arrowheads) in regions 0 to 8. Region 0: Omental caking (∗) and a nodule in the lesser sac (arrow) are shown. Regions 1 to 3: There are nodular diseases in the right (black arrowheads in region 1) and left (white arrowhead in region 3) subphrenic spaces. In region 2, nodular disease in the fissure for the falciform ligament (∗) and the splenic hilum (arrowhead) are shown. Region 4: Nodule in the left lumbar region (arrowhead). Region 5: Sigmoid mesocolon (∗) and peritoneal thickening in the left lower abdomen (arrowheads). Region 6: Pelvic peritoneal thickening (arrowhead) and bilateral ovarian masses are shown (four-point star). Region 7: Nodular peritoneal thickening in the right lower abdomen (arrowhead) and abdominal wall nodule (arrow). Region 8: Nodular peritoneal thickening in the right lumbar region (arrowhead).

**Supplementary Figs. S2:**
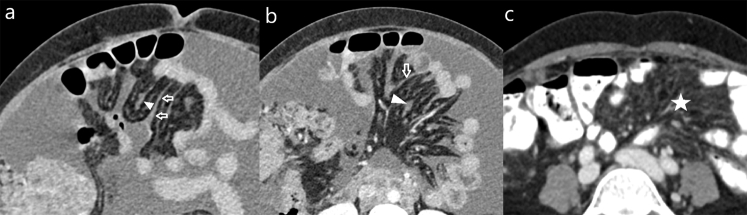
**(a-c):** Examples of CT images depicting mesenteric disease. (a, b) Mesenteric fold thickening (arrows) and nodules along the mesenteric folds (arrowhead). (c) Nodular mesenteric fat stranding (∗).

In these patients, the components of the PAUSE,^21^ were assessed. The radiological Peritoneal Cancer Index (rPCI) was calculated using the methodology described by Jacquet and Sugarbaker.^15^ Fig. 2, Supplementary Figure S1 and Supplementary Table S1 depict the CT assessment of rPCI and other components of PAUSE. rPCI was analysed as both a categorical and a continuous variable for analysis and subsequent designing of nomograms. A cut-off value of 13 was applied to dichotomise the cohort, based on prior findings by Elzarkaa et al.,^27^ which demonstrated that an rPCI >13 is associated with poorer prognosis in patients with serous epithelial ovarian cancer. The volume of upper abdominal disease was documented as the sum of the radiological PCI scores for regions 1, 2, and 3. We only marked moderate and severe ascites, which was defined as the presence of fluid in all CT slices with or without abdominal distension. Any enhancing soft tissue density nodule, either in the port site or otherwise in the abdominal wall, was marked as abdominal wall disease. Unfavourable sites were classified as U0, U1, and U2 as per prior work.^21^ In our study, we considered disease in the intersegmental fissures of the liver, lesser omentum, lesser sac, splenic hilum, recto-sigmoid colon infiltration, ureteric infiltration causing hydronephrosis and encasement of iliac vessels as U1 category of findings. Presence of thick subphrenic disease (>1 cm), encasement of vessels at the porta hepatis, biliary obstruction, supra-renal para-aortic nodes, nodules at the root of mesentery, frank small bowel obstruction from serosal disease and mesenteric tethering were considered as U2 category of findings. Patients with both U1 and U2 findings were marked as U2. Small bowel and mesenteric disease were graded as described by Yan et al.,^28^ where mesenteric nodules, mesenteric fold thickening (Supplementary Figure S2) and bowel wall thickening from bowel serosal disease were marked class 2 findings and large mesenteric masses, mesenteric tethering and bowel obstruction were marked as class 3 findings. All extra-abdominal nodes, including cardio-phrenic, inguinal nodes, mediastinal, supraclavicular and axillary nodes, were marked as extra-abdominal disease. Cardio-phrenic nodes were marked as significant and as the presence of extra-abdominal disease if the short-axis diameter was >/ = 7 mm. Extraperitoneal visceral metastases to organs such as liver, spleen, lungs, pleura, bones and adrenals were also marked as the presence of extra-peritoneal disease. These findings and the CT-based FIGO stage were documented and stored in the PACS for further analysis.

Interobserver agreement was measured to assess the reproducibility of CT PAUSE. A representative subset of 30 systemically sampled staging (n = 4) or post-neoadjuvant chemotherapy (n = 26) CT studies of ovarian carcinoma patients was used to assess interobserver agreement among six observers. The observers had a mean experience of 5 years and a range of 1–17 years in abdominal radiology.

At the investigators’ centre, imaging for all gynaecological cancers was routinely reviewed in a specialised Gynae-Oncology MDT. For this study, two radiologists documented imaging findings of consecutive ovarian cancer patients using PAUSE. The MDT—comprising surgeons, oncologists, radiologists, pathologists, and palliative care physicians—then discussed each case, made individualised treatment decisions, and recorded them in the electronic health record (EHR). Recommendations were subsequently communicated to patients in outpatient clinics and implemented by clinical teams. Routine staging laparoscopy was not performed as part of the standard MDT workflow. Patient selection was based primarily on contrast-enhanced CT interpreted within a structured PAUSE framework, supplemented by clinical assessment.

All patients were managed by MCh-trained gynaecologic oncology surgeons with 7–20 years’ experience, who oversaw care from diagnosis to surgery. Resectable cases per CT-PAUSE underwent cytoreductive surgery, while unresectable disease received platinum–taxane–based neoadjuvant chemotherapy (NACT). Responders after 3–4 NACT cycles proceeded to interval cytoreductive surgery (ICS), typically including hysterectomy, salpingo-oophorectomy, omentectomy, and peritonectomies. Non-responders received up to 6 NACT cycles; progression generally led to palliative or best supportive care. Surgical outcomes were classified by Sugarbaker criteria^29^: complete cytoreduction (CC0) was defined as no visible residual disease; incomplete cytoreduction was categorised as CC1 (residual disease <1 cm), CC2 (residual disease 1–2.5 cm) and CC3 (>2.5 cm residual disease). All perioperative details were documented in the EHR.

Preoperative ultrasound-guided omental biopsies were performed for patients with one of the following: FIGO stage 4 ovarian cancer, large volume peritoneal disease, clinically suspected non-ovarian primaries and when determining histopathological type was considered clinically important for deciding NAC, for example, low-grade serous ovarian cancers or non-epithelial ovarian cancers. Surgical specimens and biopsy specimens were reviewed by a dedicated group of pathologists with a special interest in gyneoncology and an experience of 7–12 years. Pathological staging was based on FIGO system (2014).^30^

Data collected included demographic characteristics, preoperative serum CA-125 levels, imaging findings, MDT decision and recommendations, treatment modality offered, surgical histopathology, surgical PCI, and the outcomes of surgical cytoreduction. The sources of these data are given in Supplementary Table S2.

Because published data on PAUSE in ovarian cancer were limited, the sample size was based on an unpublished retrospective audit of colorectal peritoneal metastases, in which a PAUSE score less than or equal to three was associated with complete cytoreduction (sensitivity 80%, specificity 83.3%, PPV 96%, NPV 38.4%). Using a PPV of 96%, 5% precision, and 95% confidence, the estimated sample size was 73 patients; for 99% confidence, 126 patients. The final cohort comprised 124 patients who underwent 175 CT scans.

### Statistical analysis

We performed a comprehensive statistical analysis to evaluate the association between the CT PAUSE score and MDT decision-making, as well as cytoreductive surgical outcomes. Data from the study proforma were transcribed into a structured spreadsheet according to predefined recruitment criteria. An initial assessment of data distribution was conducted to determine normality. The descriptive statistics, mean (SD) and median (IQR), were reported for the continuous variables as per the normality of the data. The numbers and percentages were used to represent categorical data. For continuous variables, independent samples t-test were used when data followed a normal distribution, while non-parametric tests (Mann–Whitney U test or Kruskal–Wallis test) were applied for skewed distributions. Associations between categorical variables—including individual components of the PAUSE score and MDT decisions—were examined using Pearson's chi-square test, Fisher's exact test, or ANOVA as appropriate. To further explore predictive relationships, multivariate logistic regression modelling was employed. A nomogram was developed based on significant variables to estimate the likelihood of specific MDT decisions. We employed a threshold of rPCI >13 only for dicotomising rPCI for initial univariate analysis based on prior literature [5] demonstrating an association with higher disease burden and less favourable prognosis. In the subsequent multivariate modelling and while designing the nomograms, we have used rPCI as a continuous variable since rPCI alone did not determine the treatment pathway. Receiver operating characteristic (ROC) curve analysis was used to evaluate the discriminative performance of the nomogram, with the concordance index (C-index) reported to assess predictive accuracy. Interobserver agreement was assessed using intraclass correlation coefficient (ICC) or Kappa statistics for individual components of PAUSE and the PAUSE-based nomograms. All statistical tests were two-sided, and a p-value of less than 0.05 was considered statistically significant. Analyses were performed using SPSS software (version 25.0, IBM Corp., Armonk, NY, USA) and RStudio version 4.4.0.

### Ethics statement

The study was approved by the Institutional Review Board (IRB) of Christian Medical College Vellore, and the IRB approval number is 14789, dated 10 August 2022. Written informed consent was obtained from all participants prior to enrolment, in accordance with the Declaration of Helsinki.

### Role of the funding source

There was no funding source for this study.

## Results

### Patient characteristics

Patient flow diagram is given in Fig. 1 and Table 1 summarises baseline characteristics. The final cohort included 124 patients, with a mean (Standard Deviation (SD)) age of 50.6 (11.2) years. A total of 175 CT scans were reviewed; 62.1% (n = 77) were initial staging scans, rest were restaging scans following neoadjuvant chemotherapy (NACT). Overall, 89% of patients received 3–6 cycles of NACT (range 2–12).

**Table 1:** Demographic characteristics.

Variable	Category	Frequency	Percentage
Mean (SD) Age (N = 124)	50.6 (11.2)	Range: 21–82 years	
Median (IQR) of First CA 125 at presentation (N = 124)	1639 (2543) U/L	Range: 56–18,093 U/L	
Initial CT FIGO (n = 124)	Stage IIIa	16	12.9
	Stage IIIb	7	5.6
	Stage IIIc	49	39.5
	Stage IVa	14	11.3
	Stage IVb	38	30.6
Median (IQR) of radiological peritoneal cancer index (rPCI)	23 (13)	Range: 1–36	
Ascites (N = 175 CT scans)	Absent/mild	98	56
	Moderate/severe	77	44
Abdominal wall (N = 175 CT scans)	Absent	172	98.3
	Present	3	1.7
Unfavourable site (N = 175 CT scans)	U0	65	37.1
	U1	49	28
	U2	61	34.8
Small bowel and mesenteric disease (N = 175 CT scans)	Class 0	78	44.5
	Class 1	21	12
	Class 2	38	21.7
	Class 3	38	21.7
Extraperitoneal disease (N = 175 CT scans)	Absent	94	53.7
	Present	81	46.3
MDT decision (n = 175)	Cytoreductive surgery	84	48
	Neoadjuvant chemotherapy (NACT)	66	37.7
	Best supportive care	25	14.2
Number of patients operated (n = 84)	Primary cytoreductive surgery	20	23.8
	Interval cytoreductive surgery	64	76.2
Surgical outcome (n = 84)	CC0	75	89.3
	CC1	7	8.3
	CC2	1	1.2
	CC3	1	1.2
Surgical histopathology (n = 84)	High grade serous carcinoma	72	85.7
	Low grade serous carcinoma	2	2.4
	Serous borderline tumour with microinvasion	2	2.4
	Mucinous carcinoma	2	2.4
	Clear cell carcinoma	2	2.4
	Endometroid adenocarcinoma	3	3.5
	Small cell neuroendocrine carcinoma	1	1.2
Pathological FIGO stage^a^ (n = 84)	ypFIGO stage 1	9	10.7
	ypFIGO stage 2	10	11.9
	p/ypFIGO stage 3	58	69
	yFIG0 stage 4	7	8.3

**Abbreviations used**: MDT, multi-disciplinary team; NACT, neoadjuvant chemotherapy; rPCI, radiological peritoneal cancer index; SD, standard deviation; IQR, interquartile range; CT, computed tomography.

a The prefix ‘yp’ denotes the pathological stage assessed at interval cytoreduction following neoadjuvant chemotherapy (NACT). In this cohort, FIGO stage IV disease was classified as FIGO IVB in six patients, due to full-thickness small bowel infiltration (n = 5) or appendiceal mucosal infiltration (n = 1), and FIGO IVA in one patient, due to persistent malignant pleural effusion.

Cytoreductive surgery was performed in 84/124 patients (67.7%). Nine patients received best supportive care and 31 were lost to follow up (Fig. 1). Of those operated, 20 (23.8%) underwent primary cytoreduction and 64 (76.2%) interval cytoreduction. Complete (CC0), optimal (CC1) and incomplete (CC2/CC3) cytoreduction was achieved in 75 (89.3%), 7 (8.3%), and 2 (2.4%) patients, respectively.

Histological distribution and FIGO stage among surgical patients is shown in Table 1.

### Imaging findings and correlation between PAUSE and MDT decision-making

**Fig. 3:**
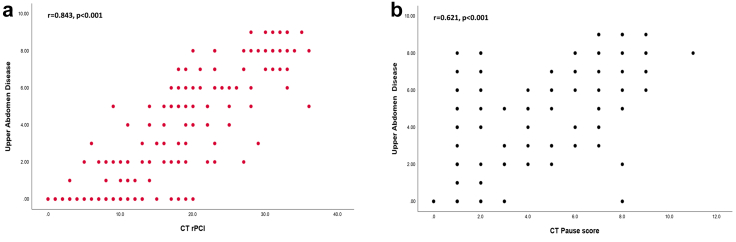
**(a, b):** (a) Scatterplot showing correlation between radiological peritoneal cancer index (rPCI) and the volume of upper abdominal disease and a strong positive correlation was identified (Pearson's r = 0.843, p < 0.001). (b) Scatterplot showing correlation between CT PAUSE score and the volume of upper abdominal disease and a moderate positive correlation was identified (Pearson's r = 0.621, p < 0.001).

Tables 1 and 2 presents the distribution of imaging findings and the results of both univariate and multivariate analyses. The mean radiological Peritoneal Cancer Index (rPCI) was 23 (SD 8) and the mean CT PAUSE score was 6 (SD 2). In the primary surgery group (n = 20), mean (SD) CT rPCI was 10 (7) versus surgical PCI 6 (4). In the interval group (n = 64), mean (SD) rPCI decreased from 23 (8) to 13 (7) post-NACT, with surgical PCI 9 (6). Multivariate analysis (Table 2) showed significant associations between most variables and MDT decisions (p < 0.001), except for abdominal wall and extraperitoneal disease. Positive correlation was observed between upper abdominal disease volume and rPCI (Pearson r = 0.843, p < 0.001; Fig. 3).

**Table 2:** Correlation between MDT-decision making with CT PAUSE score of the total 175 CTs using univariate and multivariate analysis.

MDT decisions PAUSE (N = 175 CT) components	Surgery, either primary or interval cytoreduction (N = 84) N%	NACT (N = 66) N%	Best supportive care (N = 25) N%	Univariate analysis p- value^a^	Multivariate analysis p-value^b^
P: rPCI: <13	55 (65.4)	11 (16.7)	2 (8)	<0.001	<0.001
>/ = 13	29 (34.6)	55 (83.3)	23 (92)		
A: Ascites	14 (16.6)	41 (62.1)	22 (88)	<0.001	<0.001
A: Abdominal wall	0 (0)	3 (4.5)	0 (0)	0.270	0.150
U: Unfavourable sites					
U0	52 (61.9)	13 (19.7)	0 (0)	<0.001	<0.001
U1	24 (28.5)	22 (33.3)	3 (12)		
U2	8 (9.5)	31 (47)	22 (88)		
S: Yan's CT grade (20) of small bowel and mesenteric disease					
Class 0	57 (67.8)	21 (31.8)	0 (0)	<0.001	<0.001
Class 1	14 (16.6)	6 (9)	1 (4)		
Class 2	9 (10.7)	23 (34.8)	6 (24)		
Class 3	4 (4.7)	16 (24.2)	18 (72)		
E: Extraperitoneal disease	18 (21.4)	40 (60.6)	23 (92)	0.017	0.655

Ascites were categorised as absent/mild = 0 and moderate/severe = 1. Unfavourable sites were scored as U0 = 0, U1 = 1, and U2 = 2. Small bowel and mesenteric disease were classified as Class 0 = 0, Class 1 = 1, Class 2 = 2, and Class 3 = 3.

Abbreviations used: MDT, multi-disciplinary team; NACT, neoadjuvant chemotherapy; rPCI, radiological peritoneal cancer index.

a Univariate analysis was performed for each variable individually using appropriate statistical tests, selected according to variable type, distribution, and number of groups.

b Variables with p < 0.10 in univariate analysis were included in the multivariate model. Multivariate results were adjusted for covariates retained in the final model. Statistical significance was defined as p < 0.05.

### Nomogram performance for predicting MDT decision

**Fig. 4:**
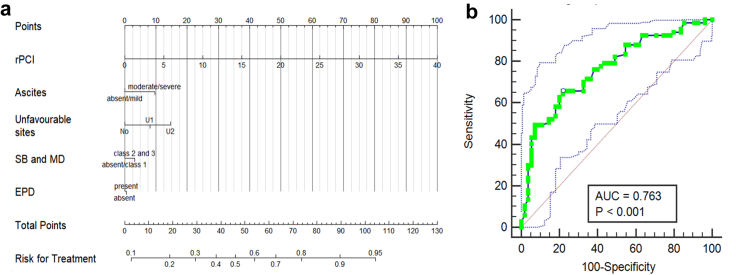
**(a, b):** (a) Nomogram from a multivariate model for multi-disciplinary team (MDT) decision-making in patients with advanced ovarian cancer using components of the CT PAUSE score. (b) Receiver Operating Characteristic (ROC) curve analysis determining the cut-off score of the above nomogram and its diagnostic performance in terms of area under the curve (AUC).

**Fig. 5:**
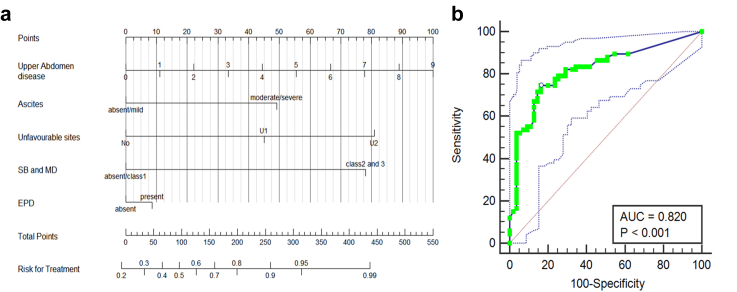
**(a, b):** (a) Nomogram from a multivariate model for MDT decision-making in patients with advanced ovarian cancer using volume of upper abdominal disease (sum of radiological peritoneal cancer index (rPCI) scores for regions 1–3) instead of total rPCI, along with the remaining components of the CT PAUSE score. (b) Receiver Operating Characteristic (ROC) curve analysis determining the cut-off score of the above nomogram and its diagnostic performance in terms of area under the curve (AUC).

A PAUSE-based logistic regression nomogram (Fig. 4) incorporating rPCI, ascites, unfavourable sites, small bowel/mesenteric disease, and extraperitoneal disease showed good discriminative ability (AUC = 0.763, 95% CI 0.677–0.835; p < 0.001). The optimal cutoff (>56 on Youden's J = 0.4385) yielded sensitivity 65.7% (95% CI 53.1–76.8) and specificity 78.1% (95% CI 65–88.2). A simplified model substituting upper abdominal disease volume for rPCI (Fig. 5) showed higher AUC (AUC = 0.820, 95% CI 0.740–0.880; p < 0.0001), with an optimal threshold >102 (Youden's J = 0.5826) providing sensitivity 74.6% (95% CI 62.5–84.5) and specificity 83.6% (95% CI 71.2–92.2).

### Interobserver agreement

Reliability analysis showed intraclass correlation coefficient (ICC) of 0.451 (95% CI 0.275–0.637; p < 0.001) for rPCI and 0.495 (95% CI 0.340–0.665; p < 0.001) for upper abdominal disease. The multi-rater Fleiss kappa (k) and its 95% CI for presence or absence of ascites, unfavourable sites, small bowel and mesenteric disease and extraperitoneal disease was 0.757 (0.664–0.849), p < 0.001; 0.396 (0.329–0.443), p < 0.001; 0.280 (0.218–0.342), p < 0.001; and 0.597 (0.504–0.689), p < 0.001, respectively. ICC (95% CI) for nomogram scores was 0.627 (0.460–0.778; p < 0.001) for Nomogram 1 (rPCI-based) and 0.710 (0.582–0.825; p < 0.001) for Nomogram 2 (upper abdominal disease-based).

## Discussion

Epithelial ovarian carcinoma is a highly lethal malignancy worldwide.^1^^,^^31^ Most patients present with extensive peritoneal spread at FIGO stage III–IV, complicating surgical outcomes.^6^^,^^32^^,^^33^ Despite advances in systemic therapies, complete cytoreduction (CC0) remains the key prognostic factor that influences survival outcomes in advanced ovarian cancer.^6^, ^7^, ^8^ Accurate preoperative imaging is therefore central to treatment planning, yet there is substantial variability in interpretation and reporting that can hinder optimal MDT decision-making.^20^^,^^21^^,^^34^^,^^35^ Structured approaches such as PAUSE for describing peritoneal disease distribution also provides a list of unfavourable disease sites that can negatively influence the outcomes of cytoreductive surgery.^21^^,^^22^ Our study evaluated the integration of PAUSE into routine workflow of ovarian cancer management.

A strength of this study is its prospective design, incorporating PAUSE scoring into real-time CT interpretation before MDT discussions. Across 175 CT scans from 124 patients with stage III/IV disease, the MDT achieved a complete cytoreduction rate of 89.3% among those selected for surgery—a result that reflects both imaging-based triage and centre-specific surgical practice. In our cohort, patients with rPCI <13 were more often directed to primary surgery (65.4%), whereas those with rPCI >13 predominantly received NACT followed by interval cytoreduction (83.3%). Although CT often underestimates PCI compared with surgical or MRI assessment,^36^^,^^37^ we observed higher rPCI values than surgical PCI in this cohort. This observation may be attributable to: (1) CT overestimation of disease burden following neoadjuvant chemotherapy due to persistent fibrotic or inflammatory changes; (2) temporal variation between imaging and surgery, with surgical PCI recorded intraoperatively and potentially differing from preoperative CT estimates, particularly in interval cytoreduction cases; and (3) conservative radiological PCI assessment during MDT triage, which may have contributed to discrepancies relative to surgical PCI.

All variables except abdominal wall and extraperitoneal disease showed significant associations with MDT decisions in multivariate analysis (p < 0.001). Unfavourable sites were seen far more frequently in the NACT and supportive-care groups than in those selected for surgery, and U2 findings were particularly predictive of non-surgical pathways. Importantly, 85% of patients who underwent cytoreduction had no small bowel or mesenteric disease on imaging, and all cases of incomplete cytoreduction demonstrated either small-bowel/mesenteric involvement or unfavourable disease sites. These findings are consistent with prior literature identifying these domains as key determinants of resectability.^20^^,^^35^^,^^38^

Overall, PAUSE showed higher discriminative performance than rPCI in predicting MDT decisions in this cohort. Upper abdominal disease volume (regions 1–3 of PCI) showed a strong correlation with rPCI (r = 0.843) and had higher discriminatory performance for predicting treatment pathways in our cohort. This aligns with earlier studies highlighting the impact of upper abdominal tumour burden—particularly disease involving the diaphragm, liver surface, porta hepatis, peripancreatic region, spleen, and lesser sac—on the feasibility of achieving optimal cytoreduction.^39^ These sites are generally included in regions 1–3 of the peritoneal cancer index framework.^15^ Inter-reader agreement was moderate for PAUSE components and substantial for nomogram scores suggesting that structured scoring tools may enhance reproducibility.

Several tools quantify peritoneal tumour burden in ovarian cancer, including tumour markers, PCI, simplified PCI systems, and laparoscopic scores.^13^^,^^23^^,^^40^, ^41^, ^42^ However, biomarkers such as CA-125 and ascites volume correlate poorly with surgical decision-making when compared with anatomical assessment.^14^^,^^40^^,^^43^^,^^44^ The intraoperative Peritoneal Cancer Index (PCI) is widely used, but its radiological counterpart (rPCI) suffers from interobserver variability and difficulty in assessing regions such as the small bowel mesentery.^13^^,^^15^^,^^18^ Simplified PCI may be easier to apply but focuses on fewer anatomical details. Laparoscopic scores, such as Fagotti,^20^ is invasive, operator-dependent, assessment may be affected by intraabdominal adhesions, and may potentially delay treatment.^45^ Staging laparoscopy is an important and widely used tool for assessing resectability in advanced ovarian cancer. Though staging laparoscopy is performed at the time of cytoreductive surgery, it is not a part of our standard MDT workflow. In this setting, CT-based PAUSE provides a non-invasive structured framework that may support multi-disciplinary decision-making. Moreover, imaging often compliments laparoscopic assessment by identifying disease in regions not easily visualised surgically, especially in patients with adhesions.^46^

MRI, especially diffusion weighted imaging (DWI) has shown higher sensitivity than CT for detecting small peritoneal deposits.^36^^,^^47^ However, its routine use is constrained by logistical limitations, competing demands on scanner availability, and the need for specialised technical and radiological expertise. Moreover, like with CT, differentiating post treatment fibrosis from small (less than 5 mm) residual nodules is challenging and limits the use of MRI as the first line imaging modality.^36^ Nevertheless, MRI is increasingly employed in selected situations, particularly for evaluating surgically occult sites.^46^

Our study has a few limitations. Most patients underwent cytoreduction after NACT, with many dropouts post-MDT, reflecting referral-centre practice where patients often return closer to home for treatment. Although confined to epithelial ovarian cancer, cohort heterogeneity from serous and non-serous subtypes limited the power to assess histology-specific differences in CT PAUSE. Abdominal wall disease, although clinically relevant, was excluded from the nomogram due to the small number of cases. The nomogram was designed to distinguish candidates for cytoreduction, not to separate patients who would benefit from NACT from those who would benefit from best supportive care. The surgical feasibility could not be pathologically confirmed in patients who did not undergo surgery, and this is an inherent limitation of imaging-based triage studies. External validation across multiple centres is essential to establish generalisability, and the low incidence of incomplete cytoreduction restricted the modelling of surgical failure. Larger, more diverse multi-institutional datasets and longitudinal studies analysing long-term progression free and overall survival outcomes are necessary to enhance predictive accuracy and evaluate the impact of PAUSE-guided MDT decision-making in unselected patient population. Despite these limitations, our findings seem to indicate the feasibility of using PAUSE in patients with advanced ovarian cancer and suggests potential relevance as a decision-support framework, similar to its application in colorectal peritoneal metastases and peritoneal mesothelioma.^24^, ^25^, ^26^

Overall, this prospective study suggests that incorporating the CT PAUSE score into MDT workflows may help promote more consistent radiological assessment and could support multi-disciplinary discussions in advanced epithelial ovarian cancer. Although imaging is only one component of patient selection, the PAUSE framework appears to be a useful structured decision-support approach. Further validation is needed to define its role in surgical triage and personalised management strategies.

## Contributors

All authors read and approved the final version of the manuscript.

*Angelin Grace Jeslin:* Study design, data collection, manuscript preparation, review.

*Shobiga Natarajan:* Study design, data collection, directly accessed and verified the data, and manuscript review.

*Sneha Hiriyanna:* Study design, data collection, and manuscript review.

*Renu Ninan:* Study design, data collection, and manuscript review.

*Goldwin Helan Cecil:* Study design, data collection, and manuscript review.

*Betty Simon*: Study design, data collection, and manuscript review.

*Anu Eapen:* Study design, interpretation of results and conclusion, and manuscript review.

*Vinotha Thomas*: Study design, interpretation of results and conclusion, and critical review.

*Anitha Thomas:* Study design, interpretation of results and conclusion, and manuscript review.

*Kripa M Varghese:* Study design, interpretation of results and conclusion, and manuscript review.

*Anjana Joel:* Study design, interpretation of results and conclusion, and manuscript review.

*Ashish Singh*: Study design, interpretation of results and conclusion, and manuscript review.

*Reka Karuppusami*: Study design, statistical analysis, contributed to statistical figures and tables, review.

*Anuradha Chandramohan*: Conception of the study idea, Study design, data collection, directly accessed and verified the data, interpretation of results and conclusion, critical review of manuscript.

## Data sharing statement

The datasets generated and/or analysed during the current study are not publicly available due to patient confidentiality but are available from the corresponding author on reasonable request.

## AI use statement

Language editing was done using Microsoft Copilot (Microsoft Corporation). These tools were employed for grammar correction and stylistic refinement; responsibility for the content rests solely with the authors.

## Declaration of interests

All authors declare that there were no competing interests

## Appendix A Supplementary data

Supplementary Tables 1 and 2

Translated Abstract Hindi

Translated Abstract Tamil
