# Supplementary material for: A decision-support tool for management of advanced epithelial ovarian cancer in a single centre in India (CT PAUSE Nomogram): a prospective Study (2022–2024)
Source: Lancet Reg Health Southeast Asia. 2026 Apr 29;49:100768. doi: 10.1016/j.lansea.2026.100768 (PMC13141603; doi:10.1016/j.lansea.2026.100768)
Supplement: Supplementary Tables 1 and 2 [file mmc1.docx]

**Supplementary table 1: Data collection form**

| **Data collection form** | | | | | |
| --- | --- | --- | --- | --- | --- |
| **Study ID** | |  | | | |
| **Age (years)** | |  | | | |
| **CA-125 level (IU/ml)** | |  | | | |
| **Date of staging CT scan** | |  | | | |
| **Indication for CT** | |  | | | |
| **Ca ovary** | | **0- no; 1 – yes** | | | |
| **Ct-FIGO stage:** | |  | | | |
| **Histological type** | | **0- not known; 1 - known** | | | |
| **Biopsy** | | **0-Not known 1-High grade serous carcinoma 2-others** | | | |
| **PAUSE score in staging CT:** | | | | | |
| **PCI** | 13 sites | | (1)<0.5 cm | (2)0.5-5 cm | (3)>5 cm |
|  | 0-central | |  |  |  |
|  | 1-right upper | |  |  |  |
|  | 2-epigastrium | |  |  |  |
|  | 3-left upper | |  |  |  |
|  | 4-left flank | |  |  |  |
|  | 5-left lower | |  |  |  |
|  | 6-pelvis | |  |  |  |
|  | 7-right lower | |  |  |  |
|  | 8-right flank | |  |  |  |
|  | 9-upper jejunum | |  |  |  |
|  | 10-lower jejunum | |  |  |  |
|  | 11-upper ileum | |  |  |  |
|  | 12-lower ileum | |  |  |  |
|  | Total | |  |  |  |
|  | rPCI score | | 0 - 0 | </=13 - 1 | >13 - 2 |

| **Ascites** | (0)absent/ mild | | (1)present and moderate/severe | | | | |
| --- | --- | --- | --- | --- | --- | --- | --- |
| **Abdominal wall** | (0)absent | | (1)present, if present midline/ paramedian | | | | |
| **Unfavourable sites of involvement** | U0 | | No unfavourable site | | | | |
|  | U1 | | Lesser omentum  Intersegmental fissures  Lesser sac  Ureters  Splenic hilum  Subphrenic disease. | | | | |
|  | U2 | | **Bile duct:** Biliary obstruction by tumour  **Subphrenic disease:** >1 cm thick subphrenic disease  **Non-regional intra-abdominal nodes**: Supracoeliac, periportal nodes  **Mesentery:** Root of mesentry, ligament of treitz, mesenteric tethering, stellate mesentery  **Bowel:** DJ flexure, upper small bowel, small bowel obstruction.  **Pelvic side wall disease:** infiltration of muscle, bones, vessels | | | | |
| **Small bowel and mesenteric disease** | (0) absent | | (1)class 1 | | (2)class 2 | | (3)class 3 |
| **Extra-peritoneal disease** | (0)absent | | (1)present, if present site:___________________ | | | | |
| **Total PAUSE**  **Score(1-10)** | Summary: rPCI -___; Ascites - ____;Abd wall - _____; U-_____; E- _______  PAUSE score: ______________ | | | | | | |
| **Date of MDT1** |  | | | | | | |
| **MDT1 decision after staging CT** | 1 – upfront surgery | | | 2- neo-adjuvant treatment | | 3- palliative chemotherapy | |
| **Treatment given** | 1 – upfront surgery | | | 2- neo-adjuvant treatment | | 3- palliative chemotherapy | |
| **If surgery** | Date of surgery:  Type of surgery: | | | TAH+BSO+omentectomy+diaphragmatic striping+lymphadenectomy | | | |
| **Outcome of upfront surgery** | CC0 (complete cytoreduction) | CC1 (optimal cytoreduction) | | CC2 (incomplete cytoreduction) | | CC3 (major tumour debulking) | |

| **Date of re-staging CT:** | | | | |
| --- | --- | --- | --- | --- |
| **y-CT-FIGO stage (re-staging CT):** | | | | |
| **CT Response: 0- complete response; 1- partial response; 2-stable disease; 3 – progressive disease** | | | | |
| **CA-125 level: IU/ml** | | | | |
| **PAUSE score in re-staging CT:** | | | | |
| **PCI [4]** | 13 sites | (1)<0.5 cm | (2)0.5-5 cm | (3)>5 cm |
|  | 0-central |  |  |  |
|  | 1-right upper |  |  |  |
|  | 2-epigastrium |  |  |  |
|  | 3-left upper |  |  |  |
|  | 4-left flank |  |  |  |
|  | 5-left lower |  |  |  |
|  | 6-pelvis |  |  |  |
|  | 7-right lower |  |  |  |
|  | 8-right flank |  |  |  |
|  | 9-upper jejunum |  |  |  |
|  | 10-lower jejunum |  |  |  |
|  | 11-upper ileum |  |  |  |
|  | 12-lower ileum |  |  |  |
|  | Total |  |  |  |
|  | rPCI score | 0 – 0 | </=13 – 1 | >13 – 2 |
| **Ascites** | (0)absent/ mild | | (1)present and moderate/severe | |
| **Abdominal wall** | (0)absent | | (1)present, if present midline/ paramedian | |
| **Unfavourable sites of involvement [2]** | U0 | | No unfavourable site | |
|  | U1 | | Lesser omentum  Intersegmental fissures  Lesser sac  Ureters  Splenic hilum  Subphrenic disease <1 cm | |
|  | U2 | | **Bile duct:** Biliary obstruction by tumour  **Subphrenic disease:** >1 cm thick subphrenic disease  **Non-regional intra-abdominal nodes**: Supracoeliac, periportal nodes  **Mesentery:** Root of mesentry, ligament of treitz, mesenteric tethering, stellate mesentery  **Bowel:** DJ flexure, upper small bowel, small bowel obstruction.  **Pelvic side wall disease**: infiltration of muscle, bones, vessels | |

| **Small bowel and mesenteric disease (Yan et al)[6]** | (0) absent | | | (1)class 1 | | (2)class 2 | | (3)class 3 |
| --- | --- | --- | --- | --- | --- | --- | --- | --- |
| **Extra-peritoneal disease** | (0)absent | | | (1)present, if present site:________________ | | | | |
| **Total PAUSE**  **Score(1-10)** | Summary: rPCI -___; Ascites - ____;Abd wall - _____; U-_____; S- ________, E- _______  PAUSE score: ______________ | | | | | | | |
| **MDT 2 date** |  | | | | | | | |
| **MDT2 decision after re-staging CT** | 1- interval cytoreduction | | | | 2- continue neo-adjuvant chemo | | 3- palliative | |
| **Treatment given** | 1- interval cytoreduction | | | | 2- continue neo-adjuvant chemo | | 3- palliative | |
| **If surgery** | Date of surgery:  Type of surgery: | | | | TAH+BSO+omentectomy+diaphragmatic striping+lymphadenectomy | | | |
| **Outcome of interval cytoreduction Sx** | CC0 (complete cytoreduction) | CC1 (optimal cytoreduction) | | | CC2 (incomplete cytoreduction) | | CC3 (major tumour debulking) | |
| **Surgical histopathology** | | |  | | | | | |

**Quick References for filling the proforma:**

**Peritoneal Cancer Index and size and extent of the primary tumour**

Largest lesion in each of 13 anatomical regions will be identified and given a score of 1-3 according to its size:

1. 0.5 cm
2. 0.5-5 cm
3. >5 cm

Sum of PCI score in all 13 anatomical regions in the peritoneal cavity gives PCI score of 0-39

1. Total PCI score < 13
2. Total PCI score >13

**Ascites:** 0-absent; 1-present

**Abdominal wall involvement:**  0-absent; 1-present

**Unfavourable sites of involvement:**

Includes anatomical sites if involved may affect the surgery or its outcome

U0- no unfavourable sites of involvement

U1- sites still amendable to complete cytoreduction although complexity of surgery is anticipated

U2-sites which are make complete cytoreduction unachievable

U0-0

U1-1

U2-2

**Small bowel and mesenteric disease:**

Small bowel-Includes kinking and distortion of the small bowel lumen, tethering of small bowel loops, eccentric or concentric mural thickening and segmental small bowel obstruction

Mesentry-includes mesenteric collections and plaque like thickening of mesentery

0-absent

1-class 1 -only ascites

2-class 2 -bowel wall thickening and mesenteric soft tissue masses

3-class 3 -complete loss of mesenteric architecture and bowel obstruction

**Extra-peritoneal disease:**

Includes retroperitoneal nodes and metastasis to the liver, lung, adrenals, bones, or the brain

0-absent

1-present

**Completeness of cytoreduction:**

CC0:complete cytoreduction-no visible disease: CC1: <1 cm; CC2: > 1 cm but < 2.5 cm; CC3: > 2.5 cm

**FIGO 2018 stage for Carcinoma Ovary:**

| Stage | FIGO 2018 |
| --- | --- |
| I | Tumour confined to ovaries or fallopian tube(s) |
| IA | Tumour limited to one ovary (capsule intact0 or fallopian tube, no tumour on ovarian or fallopian tube surface. No malignant cells in the ascites or peritoneal washings |
| IB | Tumour limited to both ovaries (capsule intact) or fallopian tubes  No tumour on ovarian or fallopian tube surface  No malignant cells in the ascites or peritoneal washings |
| IC | Tumour limited to one or both ovaries or fallopian tubes, with any of the following:  IC1 Surgical spill intraoperatively  IC2 Capsule ruptured before surgery or tumour on fallopian tube surface  IC3 Malignant calls present in the ascites or peritoneal washings |
| II | Tumour involves one or both ovaries, fallopian tubes with pelvic extension(below pelvic brim) or peritoneal cancer(Tp) |
| IIA | Extension and/or implants on the uterus and/or fallopian tubes/and/or ovaries |
| IIB | Extension to other pelvic intraperitoneal tissues |
| III | Tumour involves one or both ovaries, or fallopian tubes, or primary peritoneal cancer, with cytologically or histologically confirmed spread to the peritoneum outside the pelvis and/or metastasis tot the retroperitoneal lymphnodes |
| IIIA | Metastasis to the retroperitoneal lymphnodes with or without microscopic peritoneal involvement beyond the pelvis |
| IIIA1 | Positive retroperitoneal lymphnodes only(cytologically or histologically proven)  IIIA1(1)-metastasis ≤ 10 mm in greatest dimension (note this is tumour dimension and not lymph node dimension)  IIIA1(2)-metastasis >10 mm in greatest dimension |
| IIIA2 | Microscopic extrapelvic(above the pelvic brim) peritoneal involvement with or without positive retroperitoneal lymph nodes |
| IIIB | Macroscopic extraperitoneal metastasis beyond the pelvic brim ≤ 2 cm in greatest dimension, with or without metastasis to the retroperitoneal lymphnodes |
| IIIC | Macroscopic peritoneal metastasis beyond the pelvic brim > 2 cm in greatest dimension, with or without metastasis to the retroperitoneal lymphnodes |
| IV | Distant metastasis excluding peritoneal metastasis |
| IVA | Pleural effusion with positive cytology |
| IVB | Metastasis to extra abdominal organs ( including inguinal lymphnodes outside of abdominal cavity) |

**Supplementary table 2: Sources of data and methods of assessment.**

| Variable of interest | Source of data | Method of assessment |
| --- | --- | --- |
| Age | CWS | - |
| CA- 125 | CWS | - |
| Biopsy and histopathology | CWS | - |
| CT FIGO stage | PACS | FIGO staging |
| CT rPCI calculation | PACS | Based on Sugarbaker et al |
| CT PAUSE score | PACS | - |
| MDT decision | CWS | - |
| Treatment given- NACT/ ICS/ Palliative | CWS | Documentation in CWS |
| Surgical outcome | CWS | Documentation in CWS (Post-op notes following upfront/ interval cytoreductive surgery) |

*CSW – clinical workstation; PACS – picture achieving and communication system; rPCI – radiological PCI; MDT – multi disciplinary team; NACT – Neoadjuvant chemotherapy; ICS – interval cytoreductive surgery
