# Supplementary material for: A decision-support tool for management of advanced epithelial ovarian cancer in a single centre in India (CT PAUSE Nomogram): a prospective Study (2022–2024)
Source: Lancet Reg Health Southeast Asia. 2026 Apr 29;49:100768. doi: 10.1016/j.lansea.2026.100768 (PMC13141603; doi:10.1016/j.lansea.2026.100768)
Supplement: Translated Abstract Hindi [file mmc2.docx]

**शीर्षकः** CT PAUSE नोमोग्रामः उन्नत उपकला डिम्बग्रंथि कैंसर के बहु-विषयक प्रबंधन के लिए एक निर्णय-सहायता उपकरण ।

**लघु शीर्षकः** CT PAUSE - आधारित एमडीटी सहायता

**सारांश**

**पृष्ठभूमि:**

उन्नत एपिथीलियल डिम्बग्रंथि कैंसर (एडवांस्ड एपिथीलियल ऑवेरियन कैंसर) में सटीक प्री-ऑपरेटिव इमेजिंग आवश्यक है, जहां पूर्ण साइटोरिडक्शन सबसे मजबूत पूर्वानुमान कारक बना हुआ है । यह अध्ययन, एक संरचित डोमेन-आधारित रिपोर्टिंग उपकरण-सर्जिकल योजना और एमडीटी निर्णय लेने में इसकी उपयोगिता के लिए, CT PAUSE स्कोर का मूल्यांकन करता है ।

**विधिः**

इस संभावित क्रॉस-सेक्शनल अध्ययन में, फिगो III/IV स्टेज वाले डिम्बग्रंथि के कैंसर के 124 रोगियों के 175 कंट्रास्ट-एनहांस्ड सीटी स्कैन किए गए। PAUSE घटक-पेरिटोनियल कार्सिनोमैटोसिस इंडेक्स, एसाइट्स/पेट की दीवार के रोग, प्रतिकूल साइट्स, छोटी आंत्र/आंत्रों की झिल्ली से संबंधित (मेसेंटेरिक) रोग, और अतिरिक्त-पेरिटोनियल मेटास्टेसिज-मूल्यांकन के दौरान संभावित रूप से स्कोर किए गए । 30 मामलों के सबसेट में इंटर-ऑब्जर्वर समझौते का आकलन किया गया ।

**निष्कर्ष:**

PAUSE का उपयोग करते हुए एमडीटी ट्राइएज के परिणामस्वरूप एक उच्च पूर्ण साइटोरेडक्शन दर (89.3%) हुई । पेट के ऊपरी हिस्से के रोग की मात्रा के आधार पर एक सरलीकृत नोमोग्राम की मजबूती से अंतर दर्शाने की क्षमता (एयूसी [95% सीआई] = 0.820 [0.740-0.880]) का पता चला और पूर्ण आरपीसीआई-आधारित नोमोग्राम (एयूसी [95% सीआई] = 0.763 [0.677-0.835]) के लिए एक व्यावहारिक विकल्प की पेशकश की, विशेष रूप से व्यस्त नैदानिक सेटिंग्स में । इंटर-ऑब्जर्वर सहमति दोनों नोमोग्राम स्कोर के लिए पर्याप्त थी, पेट के ऊपरी हिस्से के रोग -आधारित नोमोग्राम (आईसीसी [95% सीआई] = 0.710 [0.582-0.825] बनाम 0.627 [0.460-0.778]; p <0.001) में अत्याधिक विश्वसनियता दिखाई दी।

**व्याख्याः**

CT PAUSE स्कोर, रेडियोलॉजिकल रिपोर्टिंग को मानकीकृत करता है, एमडीटी निर्णय लेने में सुधार करता है, और उन्नत डिम्बग्रंथि के कैंसर में सर्जिकल उपचार की प्राथमिकता निर्धारित (सर्जिकल ट्राइएज) करता है । उपचार में इसके एकीकरण की आसानी और भविष्यसूचक सटीकता, नैदानिक कार्यप्रवाह में इसे व्यापक रूप से अपनाने का समर्थन करती है ।

**फंडिंगः** इस अध्ययन के लिए कोई फंडिंग स्रोत नहीं था ।

**मुख्य शब्द**

डिम्बग्रंथि का कैंसर; साइटोरिडक्टिव सर्जरी; PAUSE स्कोर; सीटी; पेरिटोनियल मेटास्टेसिज; पेरिटोनियल कार्सिनोमेटोसिज इंडेक्स; सर्जिकल ट्राइएज

**शोध का संदर्भ**

**इस अध्ययन से पहले के साक्ष्य**

डिम्बग्रंथि कार्सिनोमा आमतौर पर उन्नत पेरिटोनियल प्रसार के साथ दिखाई देता है, जहां पूर्ण साइटोरिडक्शन सबसे मजबूत प्रोग्नोस्टिक कारक है. इसलिए सटीक प्री-ऑपरेटिव इमेजिंग महत्वपूर्ण है, फिर भी सीटी रिपोर्टिंग में भिन्नता सर्जिकल योजना के लिए इसकी उपयोगिता को सीमित करती है। हाल ही में ईएसजीएआर, ईएसयूआर, पीएसओजीआई और ईएएनएम की संयुक्त सिफारिशें स्थिरता और बहु-विषयक टीम द्वारा निर्णय लेने में सुधार के लिए PROMISE और PAUSE जैसे संरचित रिपोर्टिंग उपकरणों की वकालत करती हैं । सीटी-आधारित PAUSE उपकरण पीसीआई, जलोदर, पेट की दीवार की महत्ता, प्रतिकूल साइटों, छोटी आंत्र/ छोटी आंत्र की झिल्ली से संबंधित (मेसेंटेरिक) रोग और अतिरिक्त-पेरिटोनियल मेटास्टेस को एकीकृत करता है, लेकिन डिम्बग्रंथि के कैंसर में इसके संभावित साक्ष्य काफी कम हैं ।

**इस अध्ययन की उपयोगिता**

FIGO III/IV स्टेज के डिम्बग्रंथि कैंसर वाले124 रोगियों के एक समूह में, हमने नियमित MDT योजना के दौरान CT PAUSE स्कोर लागू किया । PAUSE का उपयोग सर्जिकल उपचार की प्राथमिकता निर्धारित करने की सुविधा प्रदान करता है, एक उच्च पूर्ण साइटोरिडक्शन दर (89.3%) प्राप्त करता है । हमने आगे पेट के ऊपरी हिस्से के रोग की मात्रा के आधार पर एक सरलीकृत नोमोग्राम विकसित किया, जिसने पूर्ण आरपीसीआई-आधारित मॉडल की तुलना में मजबूत भिन्नता की क्षमता (एयूसी 0.820) और उच्च इंटर-ऑब्जर्वर विश्वसनीयता का प्रदर्शन किया । यह व्यावहारिक उपकरण भविष्यसूचक सटीकता से समझौता किए बिना व्यस्त नैदानिक सेटिंग्स के लिए एक सुव्यवस्थित विकल्प प्रदान करता है ।

**सभी** **उपलब्ध साक्ष्यों का प्रभाव**

हमारे निष्कर्ष नियमित डिम्बग्रंथि कैंसर स्टेजिंग वर्कफ़्लो में CT PAUSE के एकीकरण का समर्थन करते हैं । रेडियोलॉजिकल रिपोर्टिंग को मानकीकृत करके और एमडीटी निर्णय लेने की क्षमता को बढ़ाकर, PAUSE सर्जिकल योजना में सुधार करता है और रेडियोलॉजिस्ट एवं सर्जनों के बीच अपेक्षाओं को संरेखित करता है । सरलीकृत नोमोग्राम एक विश्वसनीय, उपयोगकर्ता के अनुकूल दृष्टिकोण प्रदान करता है, जो संरचित इमेजिंग उपकरणों के उपयोग को व्यापक बना सकता है, अंततः उन्नत डिम्बग्रंथि कैंसर में साइटोरिडक्टिव सर्जरी के लिए रोगी चयन की क्षमता को बढ़ाता है ।
