# Supplementary material for: A decision-support tool for management of advanced epithelial ovarian cancer in a single centre in India (CT PAUSE Nomogram): a prospective Study (2022–2024)
Source: Lancet Reg Health Southeast Asia. 2026 Apr 29;49:100768. doi: 10.1016/j.lansea.2026.100768 (PMC13141603; doi:10.1016/j.lansea.2026.100768)
Supplement: Translated Abstract Tamil [file mmc3.docx]

**தலைப்பு:** CT PAUSE நோமோகிராம்: மேம்பட்ட எபிடெலியல் கருப்பை புற்றுநோயின் பலதரப்பட்ட நிர்வாகத்திற்கான ஒரு முடிவு-ஆதரவு கருவி.

**இயங்கும் தலைப்புஃ** CT PAUSE- அடிப்படையிலான MDT ஆதரவு

**சுருக்கம்**

**பின்னணி:** மேம்பட்ட எபிதீலியல் ஓவரியன் புற்றுநோயில், அறுவைச் சிகிச்சைக்கு முன் மேற்கொள்ளப்படும் துல்லியமான படிமப் பரிசோதனை மிக அவசியமானதாகும்; ஏனெனில் முழுமையான சைட்டோரிடக்‌ஷன் இன்னும் முக்கியமான முன்னறிவிப்பு காரணியாகத் திகழ்கிறது. இந்த ஆய்வு, அறுவைச் சிகிச்சைத் திட்டமிடலும் பல்துறை மருத்துவக் குழு (MDT) முடிவெடுத்தலிலும் CT PAUSE மதிப்பெண் எவ்வளவு பயனுள்ளதாக உள்ளது என்பதைக் கண்காணிக்கிறது.

**முறைகள்:** இந்த வருங்கால குறுக்கு வெட்டு ஆய்வில், FIGO நிலை III/IV கருப்பை புற்றுநோயால் பாதிக்கப்பட்ட 124 நோயாளிகள் 175 மாறுபட்ட-மேம்படுத்தப்பட்ட CT ஸ்கேன்களுக்கு உட்படுத்தப்பட்டனர். PAUSE கூறுகள்-பெரிடோனியல் கார்சினோமாடோசிஸ் குறியீடு, அசைட்டுகள்/வயிற்று சுவர் நோய், சாதகமற்ற தளங்கள், சிறிய குடல்/மெசென்டெரிக் நோய் மற்றும் எக்ஸ்ட்ரா-பெரிடோனியல் மெட்டாஸ்டேஸ்கள்-மதிப்பீட்டின் போது வருங்கால மதிப்பெண் பெற்றன. 30 வழக்குகளின் துணைக்குழுவில் இன்டரோப்சர்வர் ஒப்பந்தம் மதிப்பீடு செய்யப்பட்டது.

**கண்டுபிடிப்புகள்:** PAUSE ஐப் பயன்படுத்தி MDT முக்கோணத்தின் விளைவாக அதிக முழுமையான சைட்டோரெடக்ஷன் விகிதம் (89.3%) ஏற்பட்டது. மேல் வயிற்று நோய் அளவை அடிப்படையாகக் கொண்ட ஒரு எளிமைப்படுத்தப்பட்ட நோமோகிராம் வலுவான பாகுபாடு திறனை நிரூபித்தது (AUC [95% CI] = 0.820 [0.740-0.880]) மற்றும் முழு RPCI-அடிப்படையிலான நோமோகிராமுக்கு (AUC [95% CI] = 0.763 [0.677-0.835]) ஒரு நடைமுறை மாற்றீட்டை வழங்கியது, குறிப்பாக பிஸியான மருத்துவ அமைப்புகளில். இன்டரோப்சர்வர் ஒப்பந்தம் இரண்டு நோமோகிராம் மதிப்பெண்களுக்கும் கணிசமாக இருந்தது, மேல் வயிற்று நோய் அடிப்படையிலான நோமோகிராமில் (ICC [95% CI] = 0.710 [0.582-0.8

**விளக்கம்:**  CT PAUSE மதிப்பெண் கதிரியக்க அறிக்கையிடலை தரநிலைப்படுத்துகிறது, MDT முடிவெடுப்பதை மேம்படுத்துகிறது, மேலும் மேம்பட்ட கருப்பை புற்றுநோயில் அறுவை சிகிச்சை தரிசாக்கத்தை(triage) மேம்படுத்துகிறது. அதன் எளிதான ஒருங்கிணைப்பு மற்றும் முன்கணிப்பு துல்லியம் மருத்துவ பணிப்பாய்வுகளில் அதன் பரந்த ஏற்றத்தை  ஆதரிக்கிறது.

நிதி: இந்த ஆய்வுக்கு நிதி ஆதாரம் எதுவும் இல்லை.

**முக்கிய வார்த்தைகள்**

கருப்பை புற்றுநோய்; சைட்டோரிடக்டிவ் அறுவை சிகிச்சை; PAUSE மதிப்பெண்; CT; பெரிடோனியல் மெட்டாஸ்டேஸ்கள்; பெரிடோனியல் கார்சினோமாடோசிஸ் குறியீடு; அறுவை சிகிச்சை சோதனை

**ஆராய்ச்சி சூழல்**

**இந்த ஆய்வுக்கு முந்தைய சான்றுகள்**

கருப்பை புற்றுநோய் பொதுவாக மேம்பட்ட பெரிடோனியல் பரவலுடன் முன்வைக்கிறது, அங்கு முழுமையான சைட்டோரிடக்ஷன் வலுவான முன்கணிப்பு காரணியாகும். எனவே துல்லியமான அறுவை சிகிச்சைக்கு முந்தைய இமேஜிங் முக்கியமானது, ஆனால் சி. டி அறிக்கையில் மாறுபாடு அறுவை சிகிச்சை திட்டமிடலுக்கான அதன் பயன்பாட்டைக் கட்டுப்படுத்துகிறது. ESGAR, ESUR, PSOGI மற்றும் EANM ஆகியவற்றின் சமீபத்திய கூட்டு பரிந்துரைகள் நிலைத்தன்மை மற்றும் பலதரப்பட்ட குழு முடிவெடுப்பதை மேம்படுத்துவதற்காக PROMISE மற்றும் PAUSE போன்ற கட்டமைக்கப்பட்ட அறிக்கையிடல் கருவிகளை ஆதரிக்கின்றன. CT-அடிப்படையிலான PAUSE கருவி PCI, அசைட்டுகள், வயிற்று சுவர் ஈடுபாடு, சாதகமற்ற தளங்கள், சிறுகுடல்/மேசென்டெரிக் நோய், மற்றும் பெரிட்டோனியத்திற்கு வெளியான மெட்டாஸ்டேசிஸ் ஆகியவற்றை ஒருங்கிணைக்கிறது. ஆனால், ஓவரியன் புற்றுநோயில் இதற்கான முன்நோக்கி ஆதாரங்கள் இன்னும் குறைவாகவே உள்ளன.

**இந்த ஆய்வின் கூடுதல் மதிப்பு**

FIGO நிலை III/IV கருப்பை புற்றுநோயால் பாதிக்கப்பட்ட 124 நோயாளிகளின் வருங்கால குழுவில், வழக்கமான MDT திட்டமிடலின் போது CT PAUSE மதிப்பெண்ணைப் பயன்படுத்தினோம். PAUSE இன் பயன்பாடு அறுவை சிகிச்சை முக்கோணத்தை எளிதாக்கியது, அதிக முழுமையான சைட்டோரெடக்ஷன் விகிதத்தை (89.3%) அடைந்தது. மேல் வயிற்று நோய் அளவை அடிப்படையாகக் கொண்ட ஒரு எளிமைப்படுத்தப்பட்ட நோமோகிராமை நாங்கள் மேலும் உருவாக்கினோம், இது முழு RPCI-அடிப்படையிலான மாதிரியை விட வலுவான பாகுபாடு திறன் (AUC 0.820) மற்றும் அதிக இடைநிலை நம்பகத்தன்மையை நிரூபித்தது. இந்த நடைமுறை கருவி முன்கணிப்பு துல்லியத்தை சமரசம் செய்யாமல் பிஸியான மருத்துவ அமைப்புகளுக்கு ஒரு  எளிமையான மாற்று வழியாக உள்ளது.

**கிடைக்கக்கூடிய அனைத்து ஆதாரங்களின் தாக்கங்கள்**

வழக்கமான கருப்பை புற்றுநோய் நிலைப்படுத்தும் பணிப்பாய்வுகளில் CT PAUSE ஐ ஒருங்கிணைப்பதை எங்கள் கண்டுபிடிப்புகள் ஆதரிக்கின்றன. கதிரியக்க அறிக்கையிடலை தரப்படுத்துவதன் மூலமும், MDT முடிவெடுப்பதை மேம்படுத்துவதன் மூலமும், PAUSE அறுவை சிகிச்சை திட்டத்தை மேம்படுத்துகிறது மற்றும் கதிரியக்கவியலாளர்கள் மற்றும் அறுவை சிகிச்சை நிபுணர்களிடையே எதிர்பார்ப்புகளை சீரமைக்கிறது. எளிமைப்படுத்தப்பட்ட நோமோகிராம் ஒரு நம்பகமான, பயனர் நட்பு அணுகுமுறையை வழங்குகிறது, இது கட்டமைக்கப்பட்ட இமேஜிங் கருவிகளை ஏற்றுக்கொள்வதை விரிவுபடுத்தலாம், இறுதியில் மேம்பட்ட கருப்பை புற்றுநோயில் சைட்டோர்டக்டிவ் அறுவை சிகிச்சைக்கு நோயாளி தேர்வை வலுப்படுத்துகிறது.
